# TOP1α suppresses lateral root gravitropism in Arabidopsis

**DOI:** 10.1080/15592324.2022.2098646

**Published:** 2022-07-12

**Authors:** Hao Zhang, Ziyan Tang, Ying Zhang, Lin Liu, Dan Zhao, Xigang Liu, Lin Guo, Jingao Dong

**Affiliations:** a State Key Laboratory of North China Crop Improvement and Regulation, Key Laboratory of Hebei Province for Plant Physiology and Molecular Pathology, College of Life Sciences, Hebei Agricultural University, Baoding, China; b Ministry of Education Key Laboratory of Molecular and Cellular Biology, Hebei Collaboration Innovation Center for Cell Signaling, Hebei Key Laboratory of Molecular and Cellular Biology, College of Life Sciences, Hebei Normal University, Shijiazhuang, China; c College of Plant Protection, Hebei Agricultural University, Baoding, China; d Guangdong Provincial Key Laboratory for Plant Epigenetics, College of Life Sciences and Oceanography, Shenzhen University, Shenzhen, China; e College of Life Sciences, Hengshui University, Hengshui, China

**Keywords:** Lateral root, gravitropism, TOP1α

## Abstract

Root gravitropism is important for anchorage and exploration of soil for water and nutrients. It affects root architecture, which is one of the elements that influence crop yield. The mechanism of primary root gravitropism has been widely studied, but it is still not clear how lateral root gravitropism is regulated. Here, in this study, we found that Topoisomerase I α (TOP1α) repressed lateral root gravitropic growth, which was opposite to the previous report that TOP1α maintains primary root gravitropism, revealing a dual function of TOP1α in root gravitropism regulation. Further investigation showed that Target of Rapamycin (TOR) was suppressed in columella cells of lateral root to inhibit columella cell development, especially amyloplast biosynthesis. Our findings uncovered a new mechanism about lateral root gravitropism regulation, which might provide a theoretical support for improving agricultural production.

## Introduction

Root, the underground organ of plant, mainly functions in water and mineral salts absorption and anchoring, which can be affected by the root system structure. In Arabidopsis, the root system is composed of a primary root (PR) developed from radicle and lateral roots (LRs), which are mainly developed from primary root under the coordination of internal and external factors. LR increases the volume of soil reached by the root, and what is more, the angle formed by PR and LR affects the architecture of LR, which maximizes water and nutrient acquisition and mechanical support.^1^ Since root is highly plastic, LR architecture is easily affected by various environmental conditions, such as light, temperature, pH, and soil matric potential or structure. Among them, gravity is an important environmental condition as an inevitable element on earth.^2,3^

Great efforts have been made to decipher the mechanism of root gravitropism growth.^4–6^ Gravity sensing occurs primarily in the root cap, an organ that localized at the most distal part of the root tip to protect root meristem from the friction with soil throughout the elongation process.^7^ The root-cap columella cells (CCs) are large cells containing amyloplasts settled at the bottom as a result of responding to gravity, since the density of starch in amyloplast is higher than that of the surrounding cytoplasm. When root was reoriented by being rotated to 90° to generate a gravity stimulus, amyloplasts will be relocated to the new bottom of CC immediately, which is the beginning of the gravity sensing process and triggers a series of hierarchy changes in root such as cytoplasmic alkalinization and polarization of auxin efflux carriers.^7^ The relocalization of auxin efflux carriers results in the asymmetric distribution of auxin with more auxin flowing to the elongation zone, where it inhibits cell elongation and causes root bending.^7^ Morphological phenotype showed that PR grows straight downward, while LR always formed an angle with PR. The two types of roots have a specific gravitropism regulation mechanism although they share a similar mechanism to some extent. According to the characterization by Kiss et al., early formed LR could be termed type 1–6. When LR is just emerged from PR, termed type 1, amyloplast has not developed in CC. Only a few starch grains are developed in type 2 LR, which are relatively small. They are obviously visible in type 3 LR.^6^ Although the development of CC is important for gravity sensing, the regulation mechanism is still obscure.

Recently, we have reported that Topoisomerase I α (TOP1α) maintains PR gravitropism in Arabidopsis. As a type I topoisomerase, TOP1α is conserved in eukaryotes. It solves topological problems produced during multiple biological processes such as DNA replication, transcription, recombination, and chromatin remodeling by releasing the torsional stress.^8,9^ We found that TOP1α inhibits Target of Rapamycin (TOR) expression so that CCs are relatively stable to keep gravitropism, while other root cell division is promoted (like cells in meristem zone) when sucrose is produced through photosynthesis.^10^

In this article, we found that PR and LR responded differently to gravity stimulus, and what is more, the TOP1α expression level was responsible for this phenotype. The gravitropic response of LR in *top1α* was increased with abnormally developed CC compared with WT, which could be restored in complementary lines. TOR expression was increased in LR of *top1α* with earlier developed amyloplast, suggesting that TOR might participate in LR gravitropic growth regulation by controlling CC development downstream of TOP1α.

## Results

### PR and early formed LR responded differently to gravity stimulus

In Arabidopsis, LR grows horizontally after it is formed and then responds to gravity slowly forming an angle with PR. Finally, it will grow straight downward.^11^

**Figure 1.**
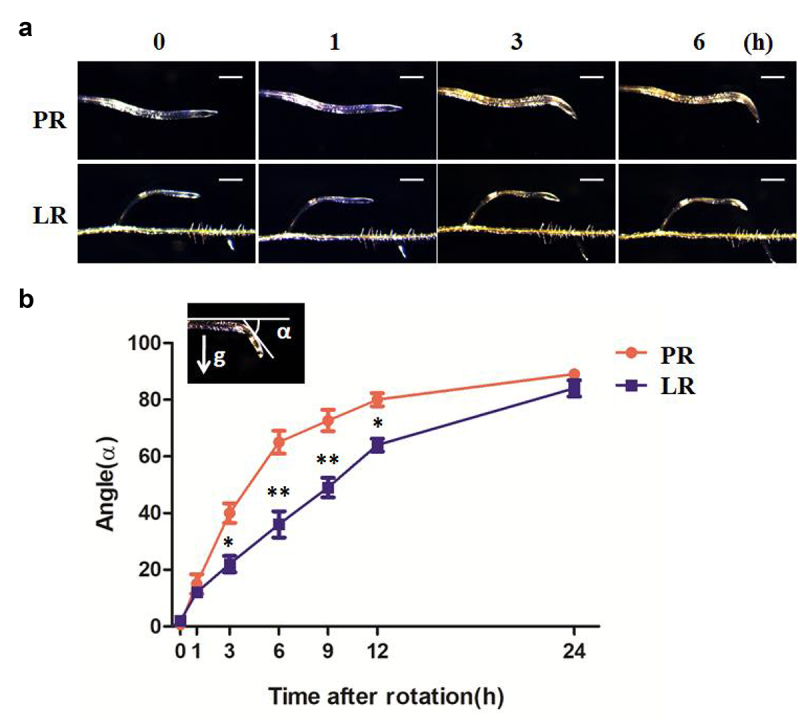
Gravitropic responses of PR and early formed LR were different in WT.

PR and LR showed different gravitropic responses in previous studies,^12,13^ and to explore the underlining mechanism, we performed gravity stimulus experiment on PR and early formed LR of wild type. By rotating root to 90°, we found that both PR and LR grew toward the gravity direction, but LR responded much slower than PR (Figure 1a, b). Statistical analysis of the bending angle showed that LR needs longer time elapse after rotation to show the same appearance similar to PR (Figure 1b). This result is consistent with previous findings, suggesting different mechanisms of gravitropism between PR and LR.

### TOP1α suppressed gravitropic growth of early formed LR

**Figure 2.**
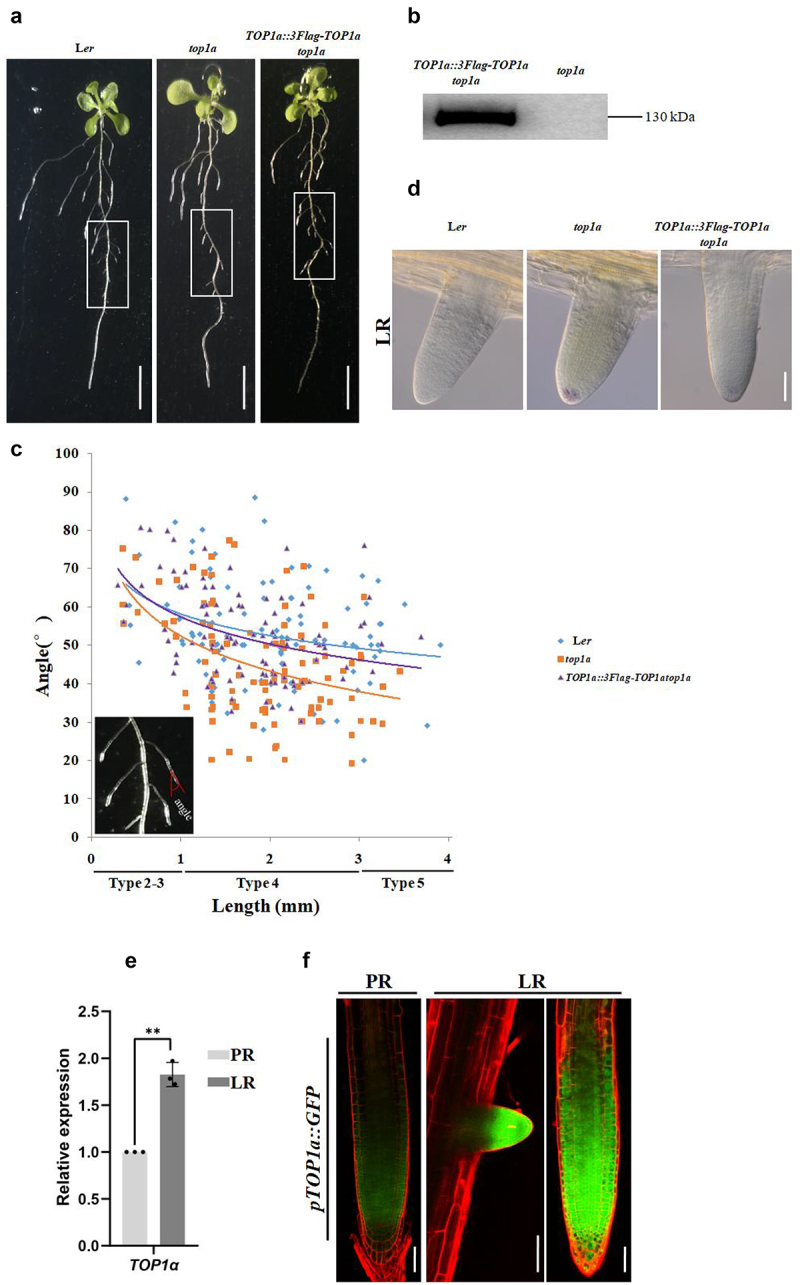
TOP1α-suppressed gravitropic growth of early formed LR.

Previously, we have reported that the PR gravitropism in *top1α* was impaired, and we further examined its LR gravitropism and found that the early formed LR of *top1α* grew more downward than WT (Figure 2a), suggesting TOP1α that might be involved in regulating gravitropic growth of early formed LR. Positive transgenic lines with single-copy transformed gene were screened and identified by Western blot (Figure 2b). We found that the LR phenotype of top1α was complemented in the *TOP1*α::*3Flag-TOP1*α *top1α* line (Figure 2a). Statistical analysis of the angle formed between PR and early formed LR with different root lengths in WT showed that the angle became smaller as LR grows. While in *top1α*, there was not much difference in the angle at the beginning, it became much smaller as LR grew compared with WT, suggesting that the early formed LR was more sensitive to gravity. The angle in the *TOP1*α::*3Flag-TOP1*α *top1α* complementary line was similar to that in WT (Figure 2c). As CC is the root cell that senses gravity, its development is one of the reasons affecting root gravitropism. We used Lugol’s solution to stain starch granules in CC of type 2 LR and found that different from early formed LR in WT with only a few starch granules in CC, starch granules had already been developed in *top1α* CC, which was much less in the *TOP1*α::*3Flag-TOP1*α *top1α* line (Figure 2d). These results were not consistent with the previous study that TOP1α promoted PR gravitropism, so we wondered if the expression of *TOP1α* in PR and LR was different in the two types of roots. We found that *TOP1α* was expressed higher in LR than PR by RT-qPCR (Figure 2e). Using the reported transgenic line *pTOP1α::GFP*, we found that although the expression level of *TOP1α* was higher in LR, the expression pattern was similar to PR (figure 2f). These results showed that TOP1α suppressed gravitropic growth of early formed LR, which was different from its promoting function in PR gravitropism.

### TOR was involved in the regulation of gravitropic growth of early formed LR by TOP1α

**Figure 3.**
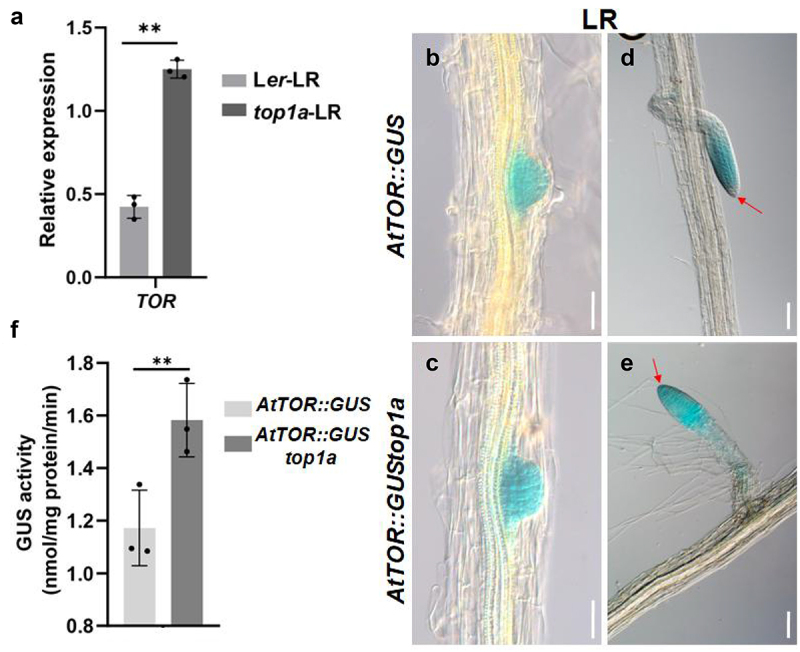
TOP1α-TOR module regulates early gravity response of LR.

We have found that TOR was repressed by TOP1α to keep the gravitropic response.^1^. To determine if TOR is involved in TOP1α-regulated gravitropic growth of early formed LR, we examined the expression of *TOR* in early formed LR of WT and *top1α*. We found that *TOR* was increased in early formed LR of *top1α* than that of WT by RT-qPCR. GUS staining results also showed the increase of *TOR* in early formed LR of *top1α* using the reported transgenic plant *AtTOR::GUS* (Figure 3 b-e); what is more, *TOR* was over-expressed in the most distal region of type 4 LR (Figure 3 d and e), although it is not obvious in emerged LR (Figures 3 B and c). GUS activity analysis showed similar results as GUS staining results. These results showed that TOR might also participate in TOP1α-regulated gravitropism of early formed LR.

## Discussion

TOP1α is involved in many biological processes including plant development. Previously, we have reported that TOP1α maintains PR gravitropism by repressing TOR expression in CC.^10^ We noticed that early formed LR in top1α is more sensitive to gravity than WT. Further investigation showed that although the expression pattern of TOP1α is similar in LR and PR, the expression level of TOP1α is higher in LR than in PR. These results reminded us that when TOP1α was repressed by its inhibitor in rice, root gravitropism was enhanced.^14^ Rice has a fibrous root system with adventitious root, while Arabidopsis has a taproot system with PR and LR. Although the adventitious roots and lateral roots are developed from different organs and subject to different regulatory mechanisms, they share key elements of the genetic and hormonal regulatory networks.^15^ The opposite functions of TOP1α in PR and LR of Arabidopsis and the consistent functions of TOP1α in LR of Arabidopsis with adventitious roots of rice might be caused by the attributes of the taproot system and fibrous root system.

The molecular mechanism in Arabidopsis and rice might be different because we found that TOP1α represses TOR expression in root especially in CC. It has been shown that TOR, as an important kinase, functions in nutrient signaling and growth control.^16,17^ We have that found TOR specifically stabilized Plethora protein PLT2 to control CC development in PR,^1^ but in this article, we found that TOR controlled LR root development by controlling amyloplast development, which has not been reported before.

## Materials and methods

### Plant materials

The *Arabidopsis thaliana* ecotype Landsberg *erecta* (L*er*) was used as wild-type (WT) in this study. *TOP1α*^18^ and *AtTOR::GUS*^19^ have been previously described. All plants were cultured in greenhouse or an incubator (CU-36L4, PERCIVAL) maintained at 22°C with 16 h light/8 h dark.

### RNA extraction and real-time quantitative PCR

Lateral roots were dissected from primary root with cutting blade. Total RNA was isolated with RNAiso Plus (TakaRa, 9109). Reverse transcription was performed using a RevertAid First Strand cDNA Synthesis Kit (ThermoFisher, K1622). cDNA was synthesized by reverse transcription using an RT reaction kit (TransGen Biotech, AT311-03) according to the manufacturer’s instructions. Quantitative real-time RT-PCR was performed in three replicates using qPCR mastermix (SYBR Green) (VAZYME, Q111-02-AA) on a Bio-Rad CFX-96 Real-time PCR Detection System. Gene expression in each sample was normalized to *UBQ5* as internal control. All primers used for RT-qPCR are listed in a previous report.^10^

All real-time PCR reactions were performed in triplicate using samples derived from three independent experiments.

### Angle statistics

After the plants were stimulated by gravity, root bending was photographed and the bending angle was measured at different time points. The angle statistics were carried out using image J software.

### Confocal microscope observation

Morphological observation of root tip cells was carried out with 100 μg/mL propidium iodide (PI) solution and photographed using a laser confocal microscope (Leica LAS SP8).

### Root tip starch staining

Seedlings were harvested and dyed with Lugol’s solution for 6 minutes and then treated with 70% alcohol for 1 minute. They were observed with DIC using a laser confocal microscope (Leica LAS V4.10).

### GUS histochemical assay

GUS histochemical assay was performed as previously described.^20^ Seedlings were stained with X-Gluc solution for a certain time at 37°C, then de-stained with 70% ethanol, and analyzed using a Leica TCS SP8 Laser confocal microscope (Leica LAS V4.10).

### Statistical analysis

In the experiments involving statistical analysis, each experiment was biologically repeated at least 3 times, and the *t*-test method was used for significance analysis.

## Data Availability

Sequence data for the genes in this article can be found in the Arabidopsis Genome Initiative or GenBank/EMBL databases under the following accession numbers: *TOP1α*, AT5G55300; *TOR*, AT1G50030; and *UBQ5*, AT3G62250.

## References

[1] Zhang Y, Ma Y, Zhao D, Tang Z, Zhang T, Zhang K, Dong J, Zhang H. Genetic regulation of lateral root development. Plant Signal Behav. 2022:2081397. doi:10.1080/15592324.2022.2081397. 35642513 PMC10761116

[2] López-Bucio J, Cruz-Ramírez A, Herrera-Estrella L. The role of nutrient availability in regulating root architecture. Curr Opin Plant Biol. 2003;6(3):280–6. doi:10.1016/s1369-5266(03)00035-9. 12753979

[3] Potters G, Pasternak TP, Guisez Y, Palme KJ, Jansen MA. Stress-induced morphogenic responses: growing out of trouble? Trends Plant Sci. 2007;12(3):98–105. doi:10.1016/j.tplants.2007.01.004. 17287141

[4] Bailey PH, Currey JD, Fitter AH. The role of root system architecture and root hairs in promoting Anchorage against uprooting forces in Allium cepa and root mutants of Arabidopsis thaliana. J Exp Bot. 2002;53(367):333–340. doi:10.1093/jexbot/53.367.333. 11807137

[5] Firn RD, Digby J. Solving the puzzle of gravitropism–has a lost piece been found? Planta. 1997;203(Suppl 1):S159–163. doi:10.1007/pl00008104. 11540324

[6] Kiss JZ, Miller KM, Ogden LA, Roth KK. Phototropism and gravitropism in lateral roots of Arabidopsis. Plant Cell Physiol. 2002;43(1):35–43. doi:10.1093/pcp/pcf017. 11828020

[7] Su SH, Gibbs NM, Jancewicz AL, Masson PH. Molecular Mechanisms of Root Gravitropism. Current Biology: CB. 2017;27(17):R964–r972. doi:10.1016/j.cub.2017.07.015. 28898669

[8] Graf P, Dolzblasz A, Würschum T, Lenhard M, Pfreundt U, Laux T. MGOUN1 encodes an Arabidopsis type IB DNA topoisomerase required in stem cell regulation and to maintain developmentally regulated gene silencing. Plant Cell. 2010;22(3):716–728. doi:10.1105/tpc.109.068296. 20228247 PMC2861470

[9] Laufs P, Dockx J, Kronenberger J, Traas J. MGOUN1 and MGOUN2: two genes required for primordium initiation at the shoot apical and floral meristems in Arabidopsis thaliana. Develop (Cambridge England). 1998;125(7):1253–1260. doi:10.1242/dev.125.7.1253. 9477323

[10] Zhang H, Guo L, Li Y, Zhao D, Dong J, Liu X. *TOP1α* fine-tunes TOR-PLT2 to keep root tip homeostasis responding to sugars. Nature Plant Accept. 2022.

[11] Guyomarc’h S, Léran S, Auzon-Cape M, Perrine-Walker F, Lucas M, Laplaze L. Early development and gravitropic response of lateral roots in Arabidopsis thaliana. Philos Trans R Soc Lond B Biol Sci. 2012;367(1595):1509–1516. doi:10.1098/rstb.2011.0231. 22527393 PMC3321682

[12] Moore R, Pasieniuk J. Graviresponsiveness and cap dimensions of primary and secondary roots of ricinus communis (Euphorbiaceae). Can J Bot. 1984;62:1767–1769. doi:10.1139/b84-239. 11540790

[13] Westberg J, Odom WR, Guikema JA. Comparative assessment of the polypeptide profiles from lateral and primary roots of Phaseolus vulgaris L. J Exp Zool. 1994;269(3):223–229. doi:10.1002/jez.1402690307. 11536635

[14] Shafiq S, Chen C, Yang J, Cheng L, Ma F, Widemann E, Sun Q. DNA topoisomerase 1 prevents R-loop accumulation to modulate auxin-regulated root development in rice. Mol Plant. 2017;10(6):821–833. doi:10.1016/j.molp.2017.04.001. 28412545

[15] Bellini C, Pacurar DI, Perrone I. Adventitious roots and lateral roots: similarities and differences. Annu Rev Plant Biol. 2014;65(1):639–666. doi:10.1146/annurev-arplant-050213-035645. 24555710

[16] Liu Y, Duan X, Zhao X, Ding W, Wang Y, Xiong Y. Diverse nitrogen signals activate convergent ROP2-TOR signaling in Arabidopsis. Dev Cell. 2021;56(9):1283–1295.e1285. doi:10.1016/j.devcel.2021.03.022. 33831352

[17] Xiong Y, McCormack M, Li L, Hall Q, Xiang C, Sheen J. Glucose-TOR signalling reprograms the transcriptome and activates meristems. Nature. 2013;496(7444):181–186. doi:10.1038/nature12030. 23542588 PMC4140196

[18] Liu X, Gao L, Dinh TT, Shi T, Li D, Wang R, Guo L, Xiao L, Chen X. DNA topoisomerase I affects polycomb group protein-mediated epigenetic regulation and plant development by altering nucleosome distribution in Arabidopsis. Plant Cell. 2014;26(7):2803–2817. doi:10.1105/tpc.114.124941. 25070639 PMC4145115

[19] Menand B, Desnos T, Nussaume L, Berger F, Bouchez D, Meyer C, Robaglia C. Expression and disruption of the Arabidopsis TOR (target of rapamycin) gene. Proc Natl Acad Sci U S A. 2002;99(9):6422–6427. doi:10.1073/pnas.092141899. 11983923 PMC122964

[20] Wang JC. Cellular roles of DNA topoisomerases: a molecular perspective. Nat Rev Mol Cell Biol. 2002;3(6):430–440. doi:10.1038/nrm831. 12042765

